# Relationship between C-Reactive Protein Level and Diabetic Retinopathy: A Systematic Review and Meta-Analysis

**DOI:** 10.1371/journal.pone.0144406

**Published:** 2015-12-04

**Authors:** Jian Song, Song Chen, Xiaoting Liu, Hongtao Duan, Jiahui Kong, Zedong Li

**Affiliations:** 1 Clinical College of Ophthalmology, Tianjin Medical University, Tianjin, China; 2 Tianjin Eye Hospital, Tianjin, China; 3 Tianjin Key Laboratory of Ophthalmology and Visual Science, Tianjin, China; 4 Tianjin Eye Institute, Tianjin, China; 5 Department of Public Health, Xinxiang Medical University, Xinxiang, China; University of Bristol, UNITED KINGDOM

## Abstract

**Objectives:**

To date, the relationship between C-reactive protein (CRP) level and diabetic retinopathy (DR) remains controversial. Therefore, a systematic review and meta-analysis was used to reveal the potential relationship between CRP level and DR.

**Methods:**

A systematic search of PubMed, Embase.com, and Web of Science was performed to identify all comparative studies that compared the CRP level of two groups (case group and control group). We defined that diabetic patients without retinopathy and /or matched healthy persons constituted the control group, and patients with DR were the case group.

**Results:**

Two cross sectional studies and twenty case control studies including a total of 3679 participants were identified. After pooling the data from all 22 studies, obvious heterogeneity existed between the studies, so a subgroup analysis and sensitivity analysis were performed. Removing the sensitivity studies, the blood CRP levels in the case group were observed to be higher than those in the control group [SMD = 0.22, 95% confidence interval (CI), 0.11–0.34], and the blood CRP levels in the proliferative diabetic retinopathy (PDR) group were also higher than those in the non-proliferative diabetic retinopathy (NPDR) group [SMD = 0.50, 95% CI, 0.30–0.70].

**Conclusions:**

The results from this current meta-analysis indicate that the CRP level might be used as a biomarker to determine the severity of DR.

## Introduction

Diabetic retinopathy (DR), a very common complication of diabetes mellitus (DM), is the leading cause of visual deficits and blindness around the world [1]. DR can be divided into two stages: non proliferative diabetic retinopathy (NPDR) and proliferative diabetic retinopathy (PDR). Typical changes seen in NPDR are micro-aneurysms, intra-retinal hemorrhages, cotton-wool spots, and hard exudate. According to the severity of the lesions, NPDR can be subdivided into mild, moderate, and severe stages. With the aggravation of the disease, the disease enters into the PDR stage. In THE PDR stage, lesions such as neovascularization of retina, disc, iris or angle, pre-retinal or vitreous hemorrhages, tractional retinal detachment can be seen [2, 3]. The mechanisms include abnormal metabolic pathways, oxidative stress, and subclinical inflammation, but the specific mechanism is not fully understood at present [4, 5]. Meanwhile, some therapeutic approaches targeting inflammation such as intravitreal injections of corticosteroids or anti-VEGF agents have showed to be effective for slowing down the development of DR [6, 7]. Therefore, inflammation seems to be very important in the development of DR.

C-reactive protein (CRP) was identified in the 1930s. It is an acute-phase protein and is mainly synthesized by the liver or adipose tissue when microbial invasion or tissue injury occurs [8]. The measurement of CRP is useful in clinical practice for the diagnosis and treatment of some acute or chronic inflammatory conditions [9].

However, the role of CRP in the pathogenesis of DR is still unknown. Clinical studies that investigate the relationship between CRP level and DR have been inconclusive. Some studies suggest that CRP level is associated with DR and with the severity of the disease [10–13]. However, some studies provide different or even opposite conclusions [14–17]. A meta-analysis is required to quantitatively synthesize the relevant work assessing the association between CRP level and DR to provide a conclusion that is more robust than individual studies. To our knowledge, there has been no meta-analysis on the relationship between CRP level and DR.

## Methods

This study was reported according to the Meta-analysis of Observational Studies in Epidemiology (MOOSE) guidelines and Preferred Reporting Items for Systematic Reviews and Meta-Analyses (PRISMA) for reporting systematic reviews and meta-analyses. Study selection, data extraction, and quality assessment were completed independently by two investigators. Disagreement was resolved through discussion. If the discussion did not lead to a consensus, Professor Chen made the final decision.

### Literature search

A systematic search of PubMed, Embase.com, and Web of Science was performed up to 18 January, 2015. The following terms, adjusted for each database, were used for the search: “high sensitivity C-reactive protein” or “high-sensitivity C-reactive protein” or “C-reactive protein” or “high-sensitive C-reactive protein” or “high sensitive C-reactive protein” or “CRP” or “hsCRP” in combination with “diabetic retinopathy” or “diabetic retinopathies” or “DR”. In order to eliminate the unrelated research as far as possible, we defined key words as mentioned above must appear in the title or abstract. Taking PubMed as an example, the specific search strategy was showed in the S1 File. In addition, to include as many related studies as possible, references of the included studies were also examined. These references did not include suitable information and were therefore excluded.

### Inclusion and exclusion criteria

Studies that met the following criteria were included in this meta-analysis: (1) comparative design. The study must contain case group and control group. We defined that diabetic patients without retinopathy and /or matched healthy persons constituted the control group, and patients with DR were the case group; (2) available CRP concentration data. CRP concentration data must contain mean and standard deviation; (3) test samples from blood. The studies in which test samples were not from blood specimen (such as tissue specimen, aqueous humor) were also excluded [18, 19]; (4) written in English or Chinese.

Studies were excluded based on the following criteria: (1) lack of a DR research aim; (2) duplicate of a previous publication; (3) inclusion of other diseases that may influence the CRP levels; (4) animal experiment. This was mainly because there was no perfect animal model to simulate the pathological changes of advanced diabetic retinopathy [20].

### Data extraction and conversion

The data from all of the included studies were extracted independently by two reviewers. The basic information was extracted from each study such as first author name, publication year, study design, CRP concentration, clinical characteristics (ages, gender, body mass index(BMI)) etc. The units of the CRP concentration were different among the studies. To facilitate the statistics, all of the units were converted into μg/L. We defined that diabetic patients without retinopathy and/or matched healthy persons constituted the control group, and patients with DR were the case group. We need data of control group and case group, but the grouping of each study was not exactly the same. Most of the original research did not include data of the two groups. Therefore, we merged the original data into the control group or case group if the study did not provide corresponding data.

### Quality assessment and study stratification

There are many methods for assessing observational studies, but the Newcastle–Ottawa scale (NOS) is the most effective. Therefore, it was used for the assessment of the included studies. The NOS is composed of three parts (8 entries): selection, comparability and exposure. A quality item is given only one star for the study in selection and exposure, and a quality item is given at most two stars for the study in comparability. It is a semi-quantitative scale, and a score of 0–9 stars is assigned to each study. Studies whose scores were more than 6 stars were considered to be of relatively high quality [21].

### Statistical analysis

Standardized mean difference (SMD) and 95% confidence interval (CI) were used to compare the two groups. Statistical heterogeneity among the studies was evaluated by the chi-squared test and *p<*0.10 indicated significance. Heterogeneity was quantitatively estimated based on I^2^, which ranged from 0% to 100% (I^2^ = 0–25%: no or mild heterogeneity; I^2^ = 25–50%: moderate heterogeneity; I^2^ = 50–75%: large heterogeneity; and I^2^ = 75–100%: extreme heterogeneity). When I^2^ was larger than 50%, a random effects model was used, and, if it was less than 50%, a fixed effect model was used.

Because significant heterogeneity existed between studies, a subgroup analysis and a sensitivity analysis were performed. The subgroup analysis was performed according to the type of DM. In order to assess the impact of heterogeneous studies on the pooled estimates, we performed a sensitivity analysis. In the sensitivity analysis, we excluded individual studies serially and obtained pooled estimates from the remaining studies. This enabled us to assess whether single studies with highly heterogeneous results were affecting the overall pooled estimates. Publication bias was evaluated using funnel plot and asymmetry test for the funnel plot. The Egger's test was used to detect the asymmetry test for the funnel plot. The existence of publication bias was mainly determined by the value of *p* from Egger's test. All analyses were performed with STATA 12.0. A *p* value<0.05 was considered statistically significant, except where otherwise specified.

## Results

### Literature search

**Fig 1:**
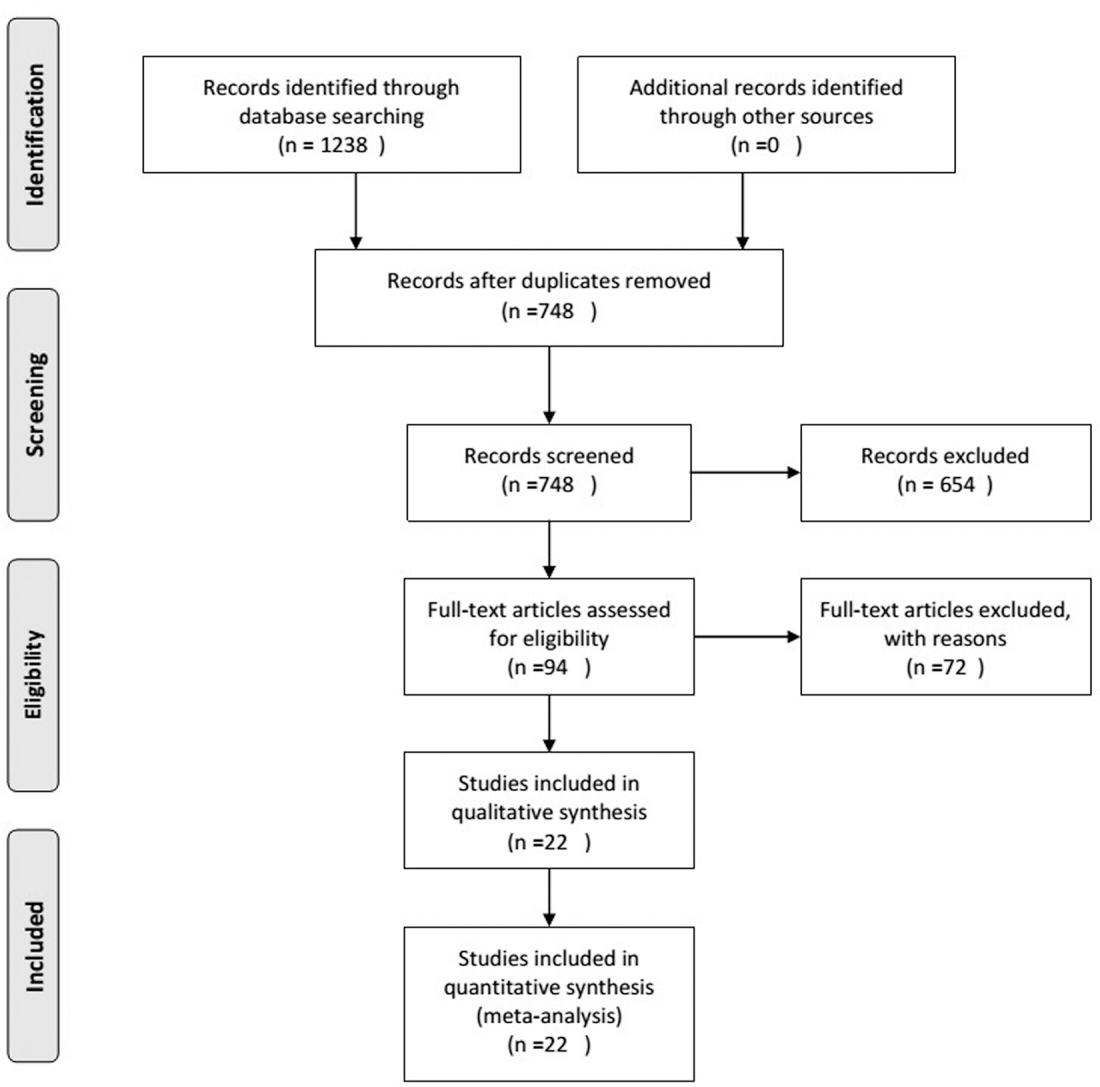
Flow chart of the literature search. A total of 1238 articles were identified from the databases, and 490 duplicates were excluded. 654 articles were excluded based on a review of the titles and abstracts, and 94 full-text articles were assessed for eligibility; 72 articles were excluded due to reasons. Finally, a total of 22 articles were included in this meta-analysis.

The study selection process is shown in Fig 1. A total of 1238 articles (PubMed 254, Embase.com 511, and Web of Science 473) were identified from the databases, and 490 duplicates were excluded using EndNote (X7). In addition, 654 articles were excluded based on a review of the titles and abstracts, and 94 full-text articles were assessed for eligibility; 72 articles were excluded due to various reasons such as being a review article or case reports, written in languages other than English or Chinese. Finally, a total of 22 [10–16, 22–36] articles were included in this meta-analysis.

### Characteristics of the studies

Two cross sectional studies [31, 32] and twenty case control studies [10–16, 22–30, 33–36] including a total of 3679 participants were identified in this meta-analysis. A total of 5 studies [27, 28, 30, 35, 36] were based on the type 1 DM including 786 participants (case group 142: control group 644), and 17 studies [10–16, 22–26, 29, 31–34] were based on type 2 DM including 2893 participants (case group 1040: control group 1853). The basic clinical information, characteristics of each study and NOS scores were presented in Tables 1 and 2. The original clinical information was shown in S1–S4 Tables.

**Table 1:** Characteristics of the studies included in the meta-analysis.

Study	Design	Location	DM type	Case group/control group	Test method	Blood	NOS(stars *)
**Blum 2012** ^**14**^	Case-control	Israel	2	NPDR+PDR/ Healthy +DM	hs-CRP	Serum	6
**Budak 2013** ^**10**^	Case-control	Turkey	2	PDR/ Healthy +DM	hs-CRP	Serum	5
**Cai 2006** ^**22**^	Case-control	China	2	NPDR+PDR/ DM	CRP	NA	4
**Chen 2010** ^**11**^	Case-control	China	2	NPDR+PDR/ Healthy +DM	hs-CRP	Serum	6
**Du 2014** ^**16**^	Case-control	China	2	NPDR+PDR/ DM	CRP	Serum	7
**Gho2014** ^**15**^	Case-control	Iran	2	NPDR+PDR/ DM	hs-CRP	NA	5
**Huang 2006** ^**23**^	Case-control	Taiwan	2	DR/Normal +DM	CRP	NA	6
**Jia 2009** ^**12**^	Case-control	China	2	NPDR+PDR/ Healthy +DM	hs-CRP	Serum	5
**Kang 2005** ^**24**^	Case-control	Korea	2	DR/ DM	hsCRP	Serum	5
**Kulkarni 2013** ^**25**^	Case-control	India	2	DR/ Healthy +DM	hsCRP	Serum	5
**Mastej 2008** ^**26**^	Case-control	Poland	2	DR/ Healthy +DM	hsCRP	Serum	6
**Mysliwiec 2008** ^**27**^	Case-control	Poland	1	DR/ Healthy +DM	CRP	Serum	6
**Mysliwska 2012** ^**28**^	Case-control	Poland	1	NPDR/DM	CRP	Serum	6
**Nayak 2006** ^**29**^	Case-control	Trinidad	2	DR/ Healthy +DM	CRP	Serum	6
**Nowak 2009** ^**30**^	Case-control	Poland	1	DR/ Healthy +DM	hsCRP	Serum	6
**Sen 2015** ^**31**^	Cross-section	India	2	DR/ Healthy +DM	hsCRP	NA	6
**Tomic 2013** ^**32**^	Cross-section	Croatia	2	NPDR+PDR / DM	CRP	NA	6
**Tsunoda 2005** ^**33**^	Case-control	Japan	2	DR/Normal +DM	hsCRP	Serum	6
**Wang 2010** ^**13**^	Case-control	China	2	NPDR+PDR/ Healthy +DM	hsCRP	Serum	6
**Yang 2014** ^**34**^	Case-control	Korea	2	DR/ Healthy +DM	hsCRP	Plasma	7
**Zorena 2007** ^**35**^	Case-control	Poland	1	NPDR / Healthy +DM	CRP	Plasma	6
**Zorena2007** ^**36**^	Case-control	Poland	1	DR/ Healthy +DM	CRP	Plasma	6

DM = Diabetes mellitus, NA = not available, CRP = C-reactive protein, hsCRP = high sensitivity C reactive protein, NPDR = Non proliferative diabetic retinopathy, PDR = proliferative diabetic retinopathy, NOS = Newcastle–Ottawa scale, Gho2014 = Gholamhossein2014, Blum 2012^14^ 14 = reference number

**Table 2:** Basic clinical information of the studies included in the meta-analysis.

Study	CRP concentration (μg/L)		Age (years)		Sex (male: female)		BMI (Kg/M^2^)	
	Case	Control	Case	Control	Case	Control	Case	Control
**Blum 2012** ^**14**^	3669±4023 (48)*	2935±3695 (48)	60.6±9.8	53.9±14.5	32:16	26:24	29.5±4.5	27.6±5.7
**Budak 2013** ^**10**^	5800±4900 (25)	4896±4186 (53)	56±9	60±6	11:14	24:29	27±6	27±6.5
**Cai 2006** ^**22**^	5285±19588 (87)	3190±7370 (103)	61.68±11.38	53.55±13.62	42:45	51:52	24.55±3.70	25.10±3.47
**Chen 2010** ^**11**^	11479±2356 (88)	6174±649 (85)	67.0±5.8	62.0±7.2	47:41	42:43	NA	NA
**Du 2014** ^**16**^	4559±2568 (39)	3330±2070 (30)	56.2±3.8	57.2±4.7	23:16	18:12	23.81±2.24	22.92±1.82
**Gho2014** ^**15**^	1649±1301 (88)	1550±1240 (92)	57.36±8.87	55.33±8.28	40:48	32:60	NA	NA
**Huang 2006** ^**23**^	4000±2300 (91)	2894±2311 (370)	NA	NA	NA	NA	NA	NA
**Jia 2009** ^**12**^	4270±2240 (79)	2449±1594 (155)	49.9±11.8	48.0±10.8	NA	NA	NA	NA
**Kang 2005** ^**24**^	1010±680 (50)	990±780 (219)	NA	NA	NA	NA	NA	NA
**Kulkarni 2013** ^**25**^	11240±2020 (50)	8030±2814 (100)	NA	NA	NA	NA	NA	NA
**Mastej 2008** ^**26**^	3510±3470 (30)	2795±3172 (42)	57.0±5.9	55.7±6.8	19:11	23:19	31.15±3.41	28.71±5.24
**Mysliwiec 2008** ^**27**^	19400±10200 (39)	11970±12559 (248)	15.15±3.55	13.41±4.08	NA	NA	20.52±3.40	19.27±2.6
**Mysliwska 2012** ^**28**^	3050±1490 (24)	2060±1650 (90)	NA	NA	11:13	NA	NA	NA
**Nayak 2006** ^**29**^	3450±3800 (30)	2550±3317 (88)	NA	NA	NA	NA	28.7±4.2	28.3±8.5
**Nowak 2009** ^**30**^	3710±2470 (41)	1160±985 (70)	42.2±11.3	39.6±11.0	17:24	28:42	20.9±2.1	22.8±3.2
**Sen 2015** ^**31**^	1340±990 (60)	380±238 (120)	55.70±9.82	55.06±11.03	NA	NA	21.00±2.31	20.8±2.97
**Tomic 2013** ^**32**^	4767±4816 (42)	3370±4140 (65)	67.40±4.87	66.31±8.31	25:27	42:23	30.48±5.26	30.77±6.06
**Tsunoda 2005** ^**33**^	705±697 (54)	765±654 (118)	65.0±9.8	62.0±7.3	NA	NA	23.6±2.7	23.1±3.0
**Wang 2010** ^**13**^	5861±2373 (87)	2589±1203 (94)	63.5±6.5	56.4±7.1	NA	NA	NA	NA
**Yang 2014** ^**34**^	1657±3362 (92)	631±614 (71)	55.5±8.6	56.1±9.2	49:43	40:31	23.2±3.7	23.8±3.9
**Zorena 2007** ^**35**^	2300±1000 (21)	1056±837 (131)	15±2	14±2.7	NA	NA	NA	NA
**Zorena2007** ^**36**^	2320±990 (17)	1060±866 (105)	15.72±3.54	13.89±3.39	NA	NA	NA	NA

CRP = C-reactive protein, BMI = body mass index, case = patients with DR, control = diabetic patients without retinopathy and /or matched healthy persons, (48)* 48 = number of participants, 3669±4023 = mean ± SD, SD = Standard Deviation, NA = not available, Gho2014 = Gholamhossein2014, Blum 2012^14^ 14 = reference number

### Meta-analysis

**Fig 2:**
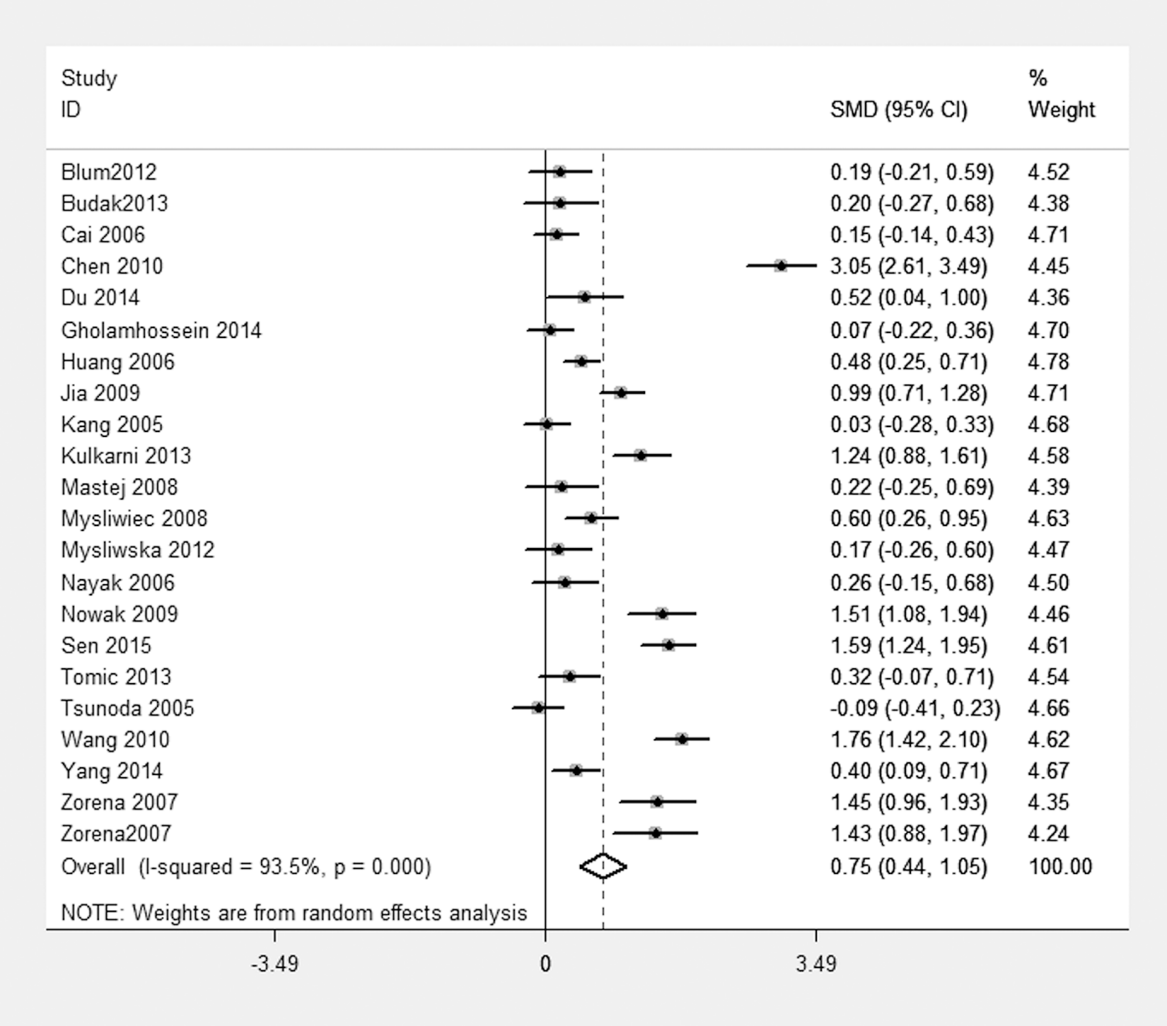
Forest plot of the 22 studies. Meta-analysis of the CRP level between case group and control group. I^2^ (I^2^ = 94.3%) was larger than 50%, a random effects model was used. SMD, standardized mean difference; CI, confidence interval.

Fig 2 shows the pooled SMD derived from all 22 studies. It indicated that the CRP level in the case group was higher than that of the control group, but heterogeneity was very obvious (I^2^ = 93.5%) [SMD = 0.75, 95% CI, 0.44–1.05].

### Subgroup and sensitivity analysis

**Fig 3:**
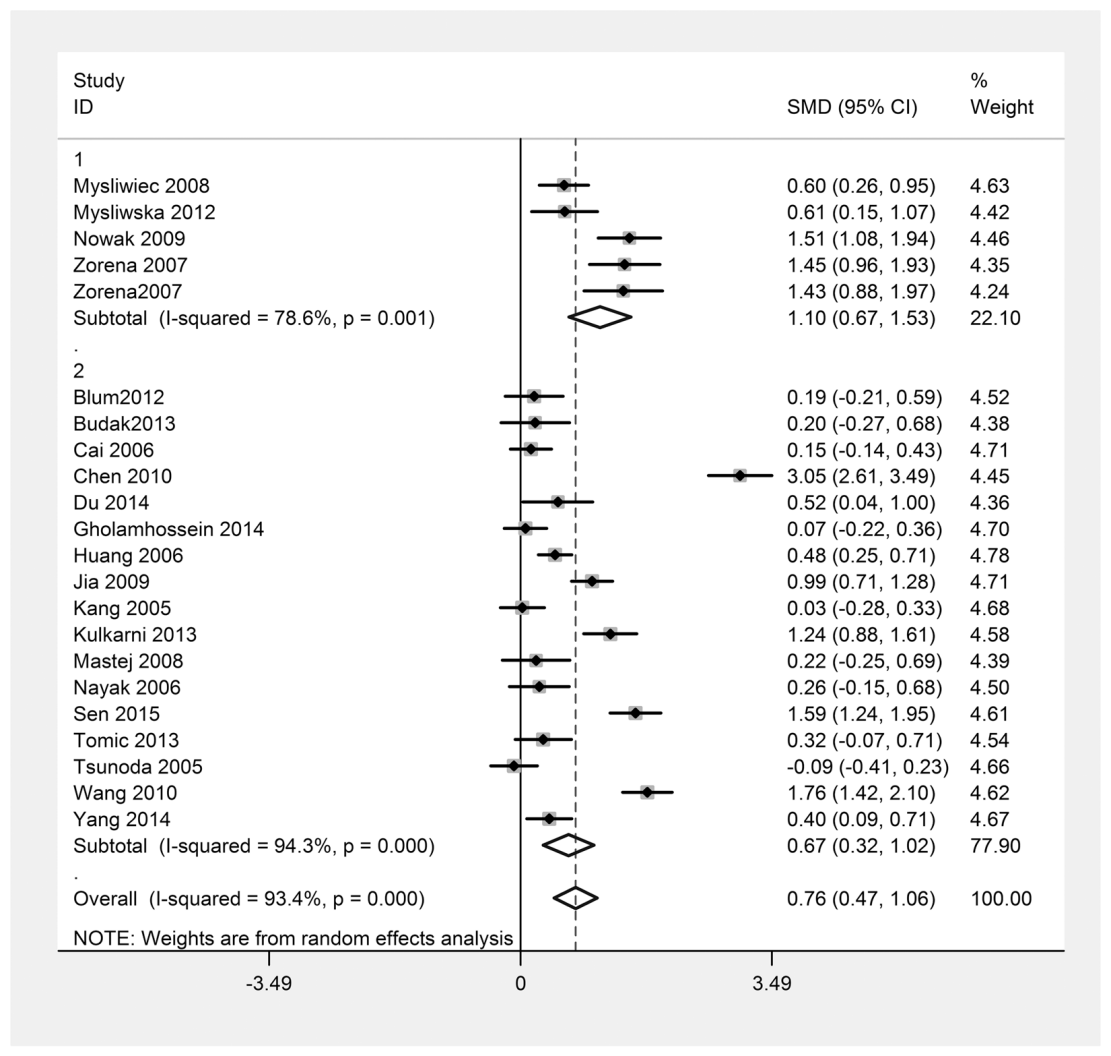
Subgroup analysis based on the type of diabetes. I^2^ (I^2^ = 78.6%, 94.3% respectively) was larger than 50%, a random effects model was used. 1, Type 1 diabetes; 2, Type 2 diabetes; SMD, standardized mean difference; CI, confidence interval.

Because there was an obvious heterogeneity among the 22 studies, it was necessary to perform subgroup and sensitivity analyses. The type of DM may be the main causes of heterogeneity, but heterogeneity was still obvious (I^2^ = 78.6% type 1 DM, I^2^ = 94.3% type 2 DM) after the subgroup analysis (Fig 3).

**Fig 4:**
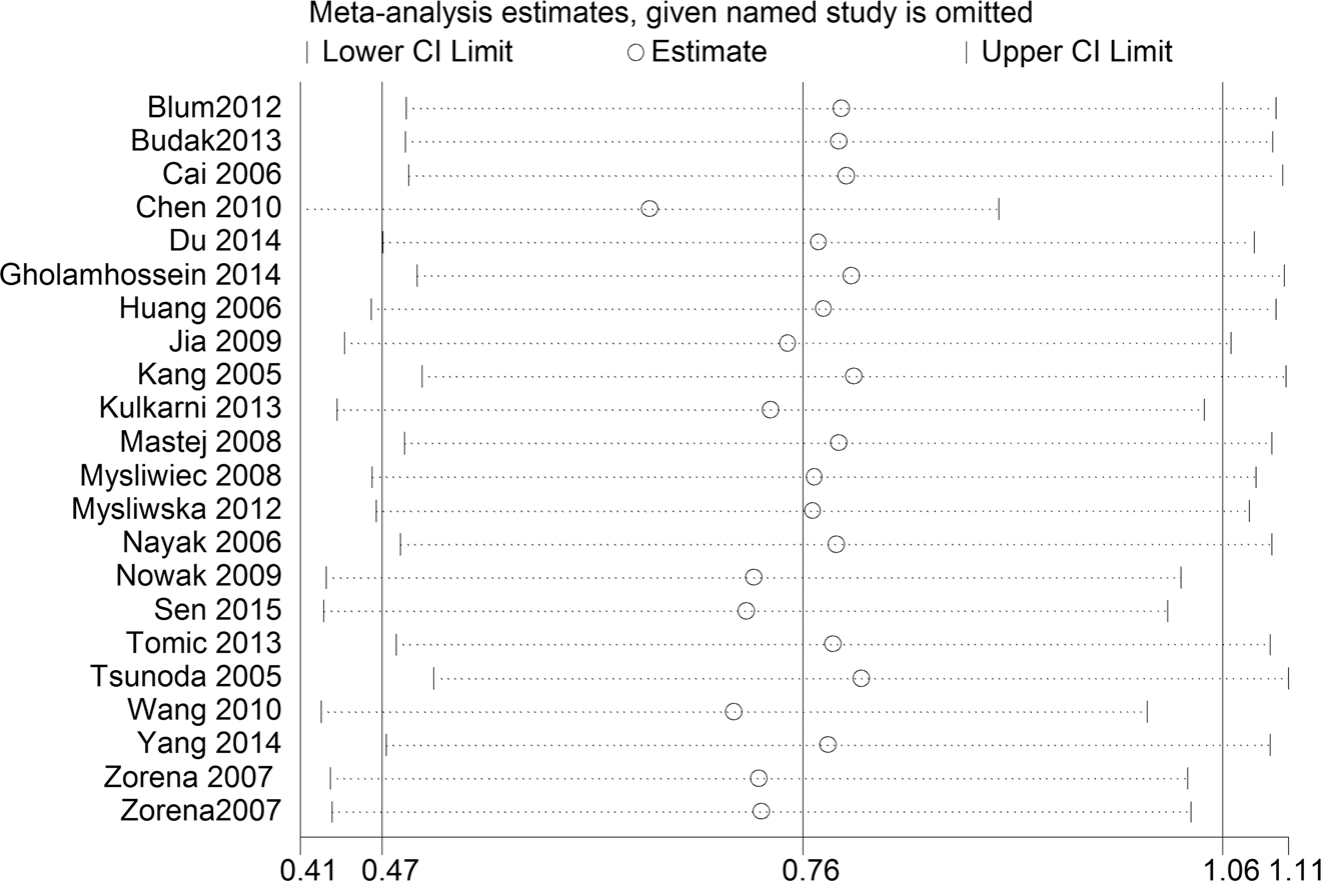
Sensitivity analysis of the 22 studies. Sensitivity analysis was performed according to omit one study in each turn. CI, confidence interval.

**Fig 5:**
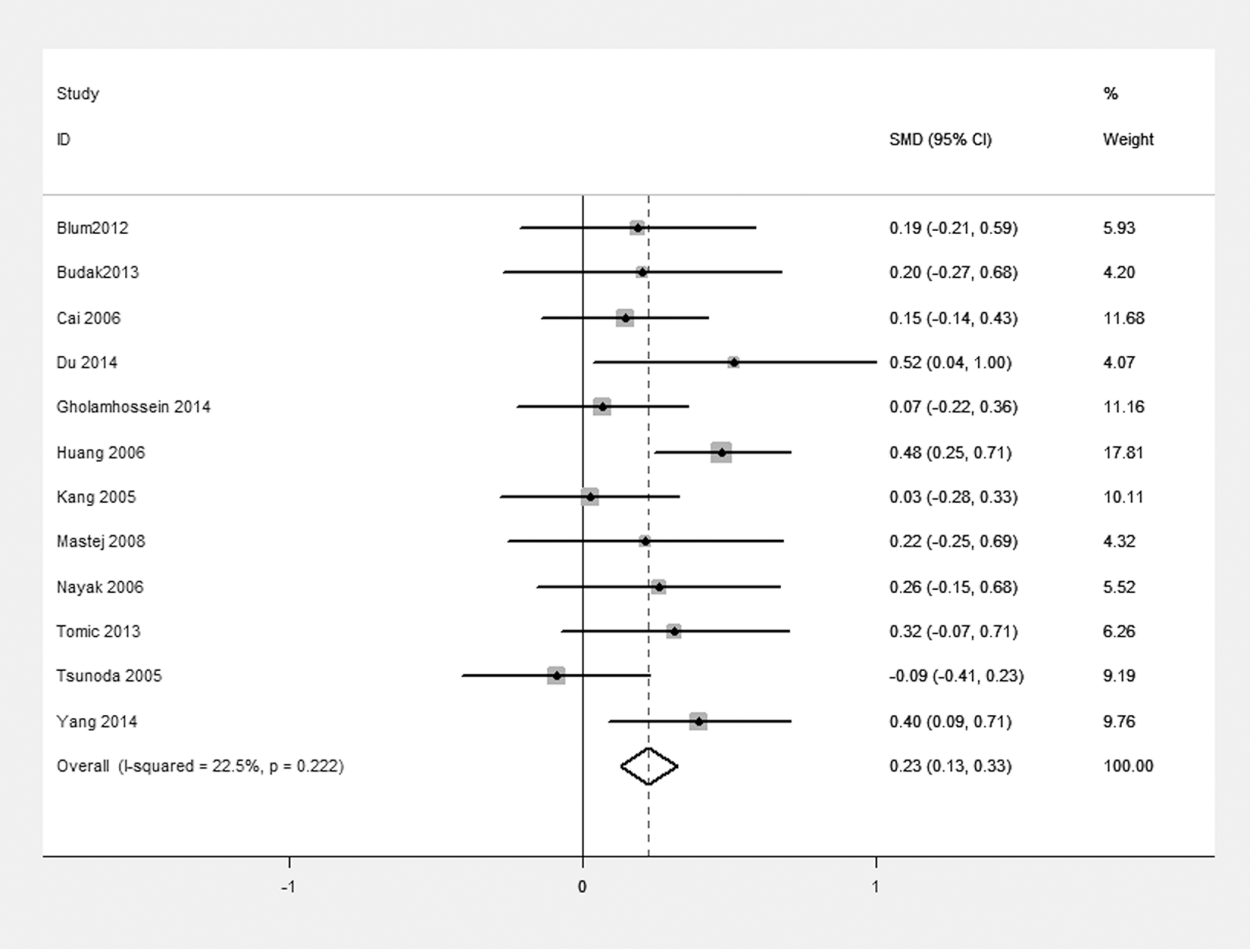
Forest plot of 12 studies after removing the sensitive studies. Meta-analysis of the CRP level between case group and control group. I^2^ (I^2^ = 22.5%) was less than 50%, a fixed effect model was used. SMD, standardized mean difference; CI, confidence interval.

A sensitivity analysis was used to evaluate the stability and reliability of the results (Fig 4). After removing the studies [11–13, 25, 27, 30, 31, 35, 36] that were contributing most to the heterogeneity, the blood CRP level in the case group was observed to be higher than that in the control group [SMD = 0.23; 95% CI, 0.13itivit (Fig 5). The results did not change substantially before and after removing the studies.

**Fig 6:**
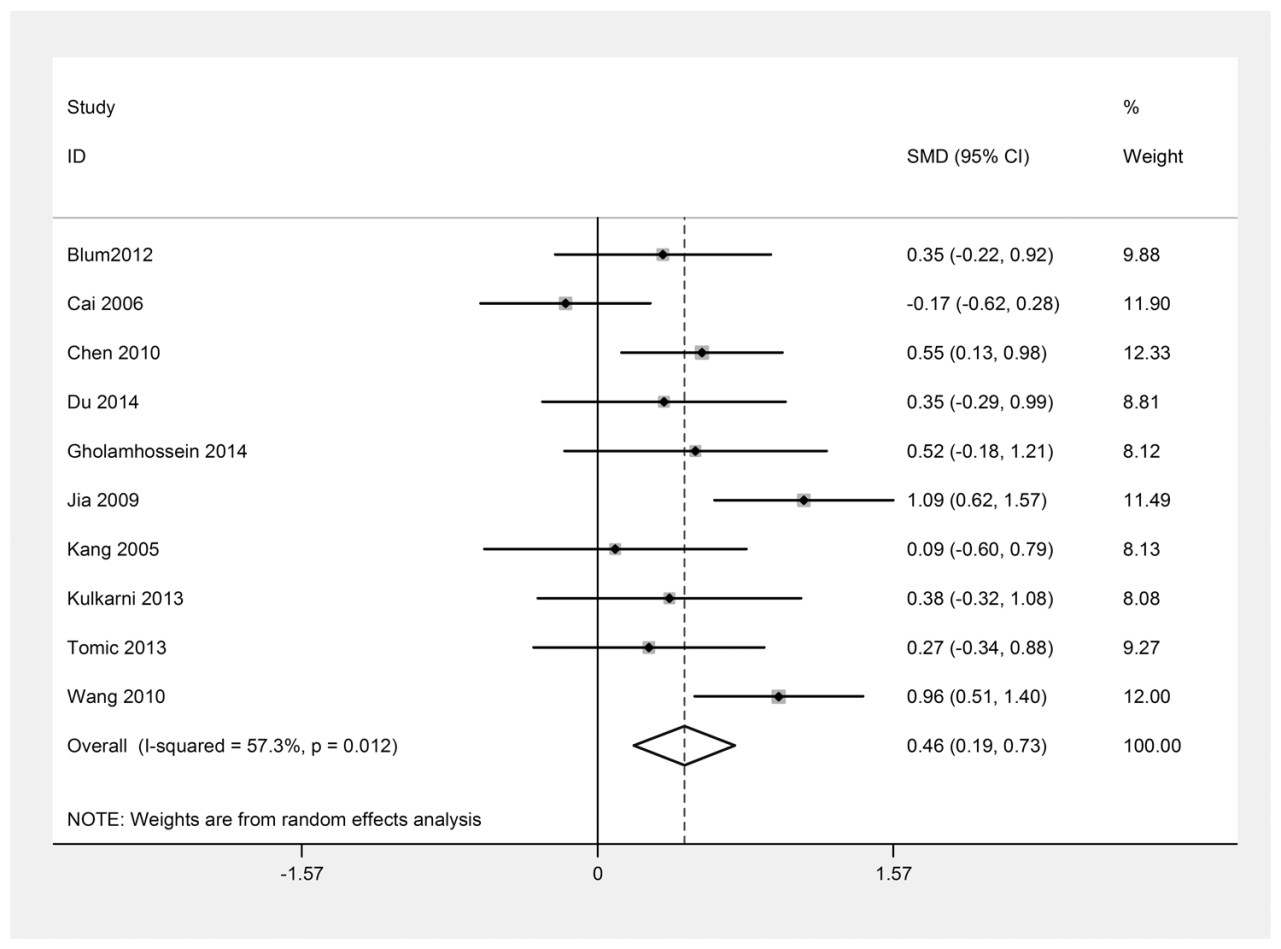
Forest plot of the 10 studies which patients with DR were divided into PDR and NPDR groups. Meta-analysis of the CRP level between the two groups. I^2^ (I^2^ = 57.3%) was larger than 50%, a random effects model was used. SMD, standardized mean difference; CI, confidence interval.

**Fig 7:**
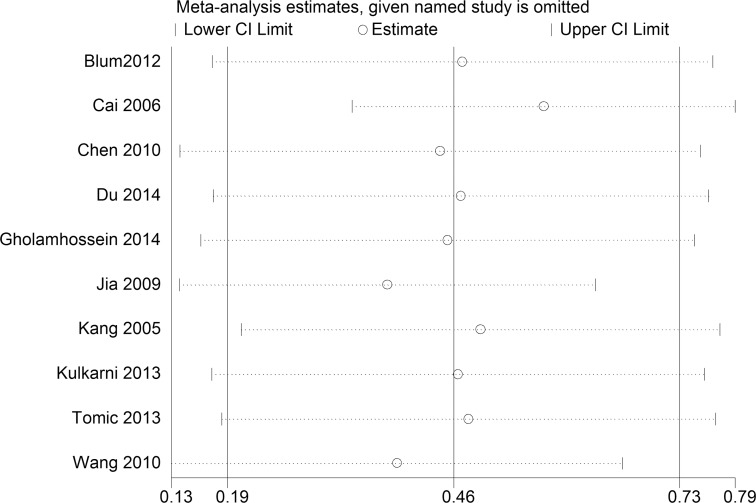
Sensitivity analysis of the 10 studies. Sensitivity analysis was performed according to omit one study in each turn. CI, confidence interval.

**Fig 8:**
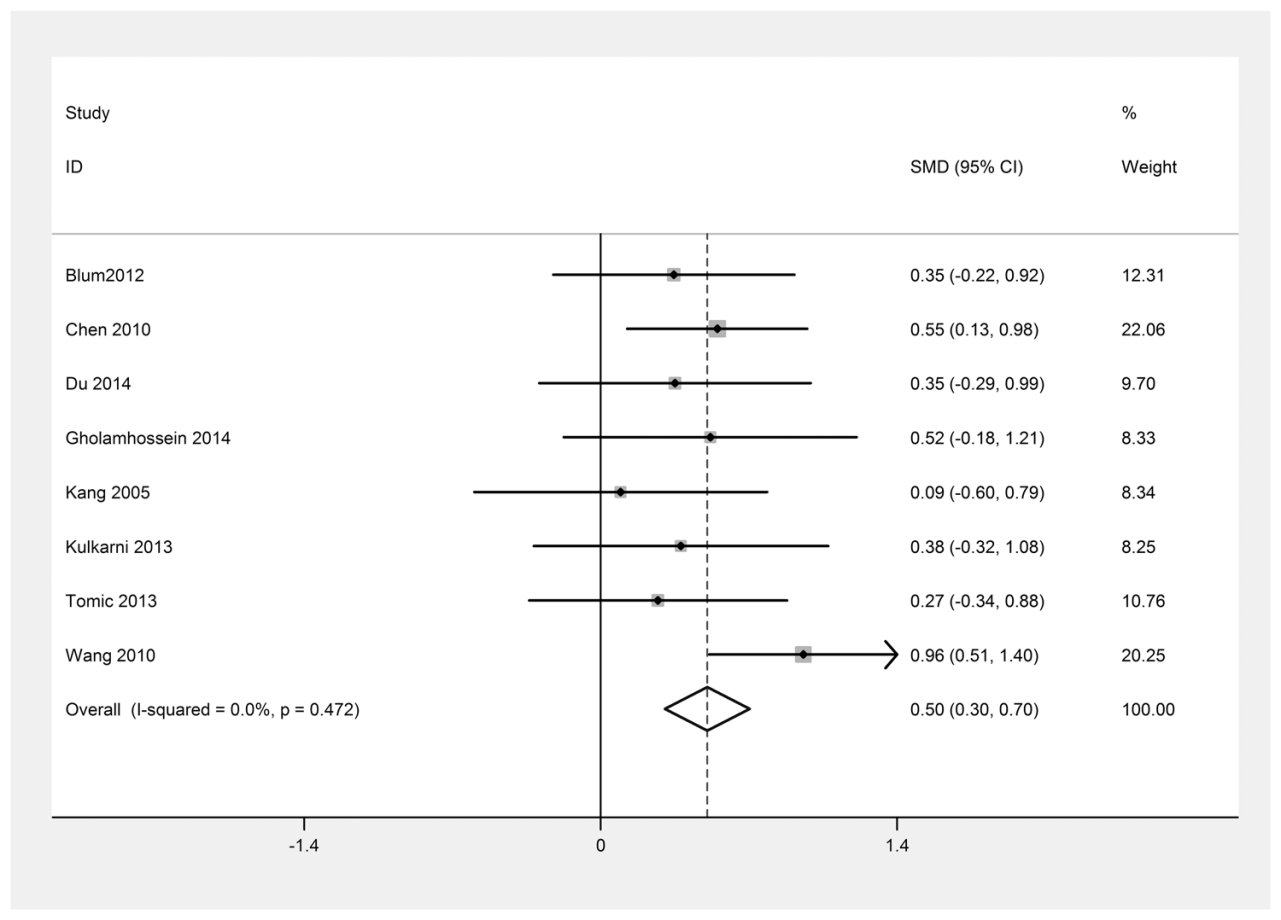
Forest plot of 8 studies after removing the sensitive studies. Meta-analysis of the CRP level between PDR group and NPDR group. I^2^ (I^2^ = 0.0%) was less than 50%, a fixed effect model was used. SMD, standardized mean difference; CI, confidence interval.

There were 10 studies [11–16, 22, 24, 25, 32] in which patients with DR were divided into PDR and NPDR groups. The differences in the CRP levels between the two groups were also analyzed to determine whether the CRP levels were related to the DR severity. The results also indicated that heterogeneity persisted (Fig 6). Therefore, a sensitivity analysis was again performed (Fig 7). After removing the studies [12, 22] that were contributing most to the heterogeneity, the blood CRP level in the PDR group was observed to be higher than that in the NPDR group [SMD = 0.50; 95% CI, 0.30–0.70] (Fig 8).

### Publication bias

**Fig 9:**
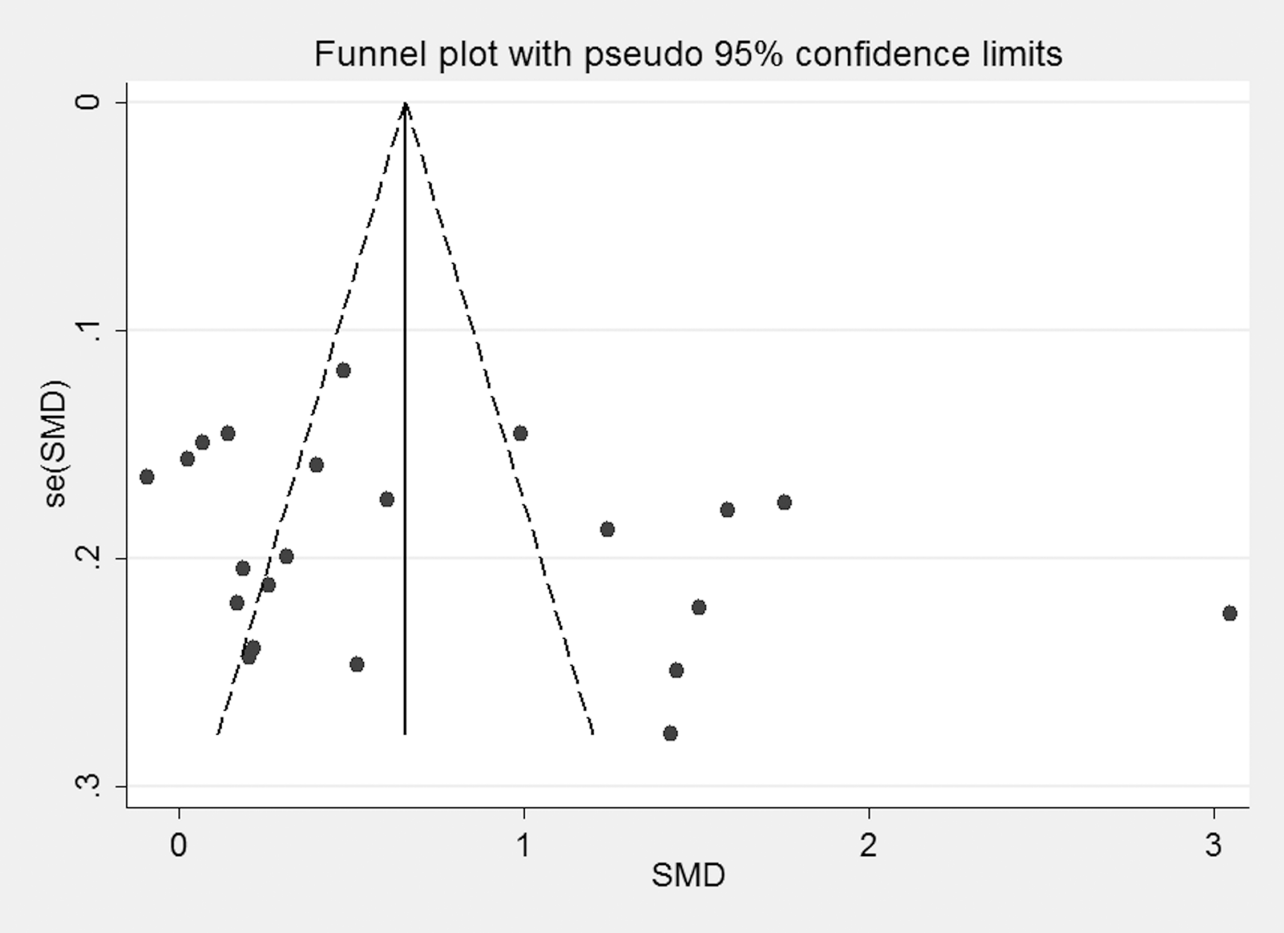
funnel plot of the 22 studies evaluating the association between CRP level and DR. CRP, C-reactive protein; DR, diabetic retinopathy; SMD, standardized mean difference; se(SMD), standard error of SMD.

Fig 9 shows the results of the Egger analysis assessing publication bias; a *p* value of 0.169 indicated there is no evidence of publication bias for the included studies.

## Discussion

This meta-analysis compared the CRP level between control group and case group. The control group was derived from diabetic patients without retinopathy and /or matched healthy persons, and the case group was derived from patients with DR. The results showed that the CRP level in the case group was higher than that in the control group, and the results were not affected by DM type. Comparing the different DR severities showed that the CRP level in the PDR group was also higher than that in the NPDR group. This result indicated that CRP level may be related to the severity of DR.

DR is a micro-vascular complication of DM, which is mainly due to the long-term high blood glucose [6]. The pathogenesis and influencing factors of DR are complex and not completely understood. Many studies have investigated the relationship between CRP level and DR, but the conclusions were not consistent. The inconsistent conclusions may be due to various reasons such as including other clinical parameters other than CRP (duration of DM and BMI), the varying definitions of DR, the study design, and the CRP detection method.

The pathogenesis and clinical characteristics differ between type 1 and type 2 DM. This could lead to inconsistent results between studies which recruited type 1 and type 2 DM participants. When we merged the 22 studies, we found that there was obvious heterogeneity. Therefore, the subgroup analysis was carried out according to the type of DM. After subgroup analysis, the heterogeneity was still obvious. It indicated that there were other factors affecting the results. Schram et al. researching the micro-vascular complications and cardiovascular disease in type 1 diabetes found that there were statistically significant differences among the three groups (diabetes without retinopathy, NPDR, and PDR) in terms of CRP concentration, but the differences disappeared after BMI values were adjusted [37]. Izuora et al. also obtained similar results [38]. At the beginning of the study, they found that there was a significant relationship between the grades of retinopathy and CRP. However, after controlling for age, duration of diabetes, sex, and BMI, the significance was lost. The duration of diabetes may also affect the study results. A study by Mysliwska et al. showed that the increase in CRP concentration was consistent with the prolonged duration of diabetes and the changes seemed to be significant based on the statistics [28]. Most researchers of the included studies also took into account the basic clinical information such as age, sex, BMI and so on. Therefore, they minimized the impact of interference factors as far as possible when they designed the studies.

According to the source of heterogeneity, it can be divided into clinical heterogeneity, methodological heterogeneity and statistical heterogeneity. The existence of heterogeneity influences the combined effect of the studies, and also the interpretation of the results of meta-analysis. Strict inclusion and exclusion criteria can help to control the heterogeneity of the studies, but, due to the presence of some potential confounding factors, there was still heterogeneity. The widely used methods to handle heterogeneity are subgroup and sensitivity analysis. Therefore, subgroup and sensitivity analysis were carried out. The NOS score of each study was relatively close, so, subgroup analysis was not carried out based on it. We attempted to improve the reliability of our study by performing a sensitivity analysis. Stepwise removal of the studies that were contributing most to the heterogeneity made no difference to the observed effect of CRP on DR. Therefore, we felt that our results were reliable.

Some studies demonstrated that CRP level in patients with PDR was higher than in NPDR patients [11, 22]. The results were consistent with the research by Mysliwska. The current meta-analysis also produced the same results.

In the detection of CRP concentration, some studies have referred to the HsCRP. In fact, HsCRP, which is higher than CRP in only the detection accuracy, can detect lower concentrations of CRP and is not a new biomarker [39]. Therefore, subgroup analysis of CRP or HsCRP was not performed when significant heterogeneity existed among the studies.

The current meta-analysis has some limitations that must be taken into account. First, because only three databases (PubMed, Embase.com, and Web of Science) were searched, some studies may have been missed. These databases are relatively well-known databases and publication bias detection indicated that there was no publication bias, so the results were credible. Second, there were fewer studies based on type 1 diabetes (only 5), which may be associated with lower morbidity. The subgroup analysis showed that the relationship between CRP and DR was not associated with the type of diabetes. However, the results need to be further proved. Third, the standard of the stage of DR was not uniform, which may affect the results. There are many classification methods for DR such as modified Airlie House classification, International Clinical Disease Severity Scale for DR and so on [40]. The latter classification is simple, practical and scientific. It is suitable for clinical application and scientific research. Therefore, we recommend using this classification method for the severity of DR. In our opinion, the results will be more credible if the researcher eliminate all kinds of interference factors as far as possible when they design the studies in the future. Finally, the present meta-analysis did not investigate the relationship between CRP and other factors that influenced the development of DR. This part of the work will be performed in follow-up studies.

In conclusion, the results of this current meta-analysis indicate that the CRP level might be used as a biomarker to determine the severity of DR. It might be beneficial to understand pathogenesis of DR. In addition, considering the limitations of this meta-analysis, large-volume, well-designed studies should be considered and conducted to validate the current results.

## Supporting Information

S1 PRISMA Checklist PRISMA Checklist. (DOC)

S1 File search strategy (PubMed). (DOCX)

S1 Table CRP concentrations in all the studies. (DOCX)

S2 Table Age in all the studies. (DOCX)

S3 Table Sex in all the studies. (DOCX)

S4 Table BMI in all the studies. (DOCX)
