# Supplementary material for: Relationship between C-Reactive Protein Level and Diabetic Retinopathy: A Systematic Review and Meta-Analysis
Source: PLoS One. 2015 Dec 4;10(12):e0144406. doi: 10.1371/journal.pone.0144406 (PMC4670229; doi:10.1371/journal.pone.0144406)
Supplement: S1 File — (DOCX) [file pone.0144406.s002.docx]

**S1 File. search strategy (PubMed)**

1. “high sensitivity C-reactive protein”[Title/Abstract]

2. “high-sensitivity C-reactive protein”[Title/Abstract]

3. “C-reactive protein”[Title/Abstract]

4. “high-sensitive C-reactive protein”[Title/Abstract]

5. “high sensitive C-reactive protein”[Title/Abstract]

6. CRP[Title/Abstract]

7. hsCRP[Title/Abstract]

8. 1 or 2 or 3 or 4 or 5 or 6 or 7

9. “diabetic retinopathy”[Title/Abstract]

10. “diabetic retinopathies”[Title/Abstract]

11. DR [Title/Abstract]

12. 9 or 10 or 11

13. 8 AND 12
