# Supplementary material for: Relationship between C-Reactive Protein Level and Diabetic Retinopathy: A Systematic Review and Meta-Analysis
Source: PLoS One. 2015 Dec 4;10(12):e0144406. doi: 10.1371/journal.pone.0144406 (PMC4670229; doi:10.1371/journal.pone.0144406)
Supplement: S1 Table — (DOCX) [file pone.0144406.s003.docx]

**CRP concentrations in all the studies**

| CRP concentration（µg/L） CRP concentration（µg/L） | | | | | | |
| --- | --- | --- | --- | --- | --- | --- |
| Study | **Healthy** | **DM** | **NPDR** | **PDR** | **Case** | **Control** |
| Blum 2012^14^ | 1659±1866（23）^#^ | 4109±4533（25） | 3005±3842（25） | 4391±4175（23） | 3669±4023（48） | 2935±3695（48） |
| Budak 2013^10^ | 3200±1100（24） | 6300±5200（29） | NA | 5800±4900（25） | 5800±4900（25） | 4896±4186（53） |
| Cai 2006^22^ | NA | 3190±7370（103） | 6360±23590（59） | 3020±4340（28） | 5285±19588（87） | 3190±7370（103） |
| Chen 2010^11^ | 5650±430（40） | 6640±410（45） | 10820±2410（42） | 12080±2160（46） | 11479±2356（88） | 6174±649（85） |
| Du 2014^16^ | NA | 3330±2070（30） | 4190±2680（23） | 5090±2380（16） | 4559±2568（39） | 3330±2070（30） |
| Gho2014^15^ | NA | 1550±1240（92） | 1580±1280（79） | 2250±1410（9） | 1649±1301（88） | 1550±1240（92） |
| Huang 2006^23^ | 2400±1800（204） | 3500±2700（166） | NA | NA | 4000±2300（91） | 2894±2311（370） |
| Jia 2009^12^ | 1860±970（72） | 2960±1840（83） | 3280±1770（39） | 6340±3530（40） | 4270±2240（79） | 2449±1594（155） |
| Kang 2005^24^ | NA | 990±780（219） | 980±47（24） | 1040±1120（12） | 1010±680（50） | 990±780（219） |
| Kulkarni 2013^25^ | 5890±1510（50） | 10170±2090（50） | 10630±1900（40） | 11390±2400（10） | 11240±2020（50） | 8030±2814（100） |
| Mastej 2008^26^ | 1260±1360（20） | 4190±3700（22） | NA | NA | 3510±3470（30） | 2795±3172（42） |
| Mysliwiec 2008^27^ | 5200±3900（85） | 15500±14000（163） | NA | NA | 19400±10200（39） | 11970±12559（248） |
| Mysliwska 2012^28^ | NA | 2060±1650（90） | 3050±1490（24） | NA | 3050±1490（24） | 2060±1650（90） |
| Nayak 2006^29^ | 2000±2000（44） | 3100±4200（44） | NA | NA | 3450±3800（30） | 2550±3317（88） |
| Nowak 2009^30^ | 570±460（35） | 1750±1020（35） | NA | NA | 3710±2470（41） | 1160±985（70） |
| Sen 2015^31^ | 460±220（60） | 300±230（60） | NA | NA | 1340±990（60） | 380±238（120） |
| Tomic 2013^32^ | NA | 3370±4140（65） | 4050±3340（19） | 5360±5770（23） | 4767±4816（42） | 3370±4140（65） |
| Tsunoda 2005^33^ | 680±570（74） | 909±760（44） | NA | NA | 705±697（54） | 765±654（118） |
| Wang 2010^13^ | 2010±850（45） | 3120±1240（49） | 4890±1660（46） | 6950±2590（41） | 5861±2373（87） | 2589±1203（94） |
| Yang 2014^34^ | 550±486（41） | 742±749（30） | NA | NA | 1657±3362（92） | 631±614（71） |
| Zorena 2007^35^ | 300±40（41） | 1400±800（90） | 2300±1000（21） | NA | 2300±1000（21） | 1056±837（131） |
| Zorena2007^36^ | 300±40（35） | 1440±830（70） | NA | NA | 2320±990（17） | 1060±866（105） |

（23）^#^ 23= number of participants, 1659±1866= mean ± SD, SD=Standard Deviation, NA = not available, CRP= C-reactive protein, DM= Diabetes mellitus, NPDR= Non proliferative diabetic retinopathy, PDR= proliferative diabetic retinopathy, Gho2014= Gholamhossein2014, Blum 2012^14^ 14= reference number,

case= patients with DR, control= diabetic patients without retinopathy and /or matched healthy persons
