# Supplementary material for: Relationship between C-Reactive Protein Level and Diabetic Retinopathy: A Systematic Review and Meta-Analysis
Source: PLoS One. 2015 Dec 4;10(12):e0144406. doi: 10.1371/journal.pone.0144406 (PMC4670229; doi:10.1371/journal.pone.0144406)
Supplement: S2 Table — (DOCX) [file pone.0144406.s004.docx]

**Age in all the studies**

| Age (years) Age (years) | | | | | | |
| --- | --- | --- | --- | --- | --- | --- |
| Study | **Healthy** | **DM** | **NPDR** | **PDR** | **Case** | **Control** |
| Blum 2012^14^ | 44.3±11.6(23)^#^ | 62.8±10.8（25） | 61.9±9.4（25） | 59.2±10.3（23） | 60.6±9.8（48） | 53.9±14.5（48） |
| Budak 2013^10^ | 61±6（24） | 59±6（29） | NA | 56±9（25） | 56±9（25） | 60±6（53） |
| Cai 2006^22^ | NA | 53.55±13.62（103） | 60.75±11.93（59） | 63.65±10.04（28） | 61.68±11.38（87） | 53.55±13.62（103） |
| Chen 2010^11^ | 59.25±6.5（40） | 64.5±6.9（45） | 65.3±5.4（42） | 68.6±5.8（46） | 67.0±5.8（88） | 62.0±7.2（85） |
| Du 2014^16^ | NA | 57.2±4.7（30 ） | 56.9±3.0（23） | 55.30±4.7（16） | 56.2±3.8（39） | 57.2±4.7（30 ） |
| Gho2014^15^ | NA | 55.33±8.28（92） | NA | NA | 57.36±8.87 (88) | 55.33±8.28（92） |
| Huang 2006^23^ | 58.2±12.2 (204) | NA | NA | NA | NA | NA |
| Jia 2009^12^ | 48.5±10.7（72） | 47.5±10.9（83） | 49.0±11.6（39） | 50.8±12.1（40） | 49.9±11.8（79） | 48.0±10.8（155） |
| Kang 2005^24^ | NA | NA | NA | NA | NA | NA |
| Kulkarni 2013^25^ | NA | NA | NA | NA | NA | NA |
| Mastej 2008^26^ | 55.4±7.5（20） | 56.0±6.2（22） | NA | NA | 57.0±5.9（30） | 55.7±6.8（42） |
| Mysliwiec 2008^27^ | 15.01±4.24（85） | 12.58±3.75（163） | NA | NA | 15.15±3.55（39） | 13.41±4.08（248） |
| Mysliwska 2012^28^ | 16.9±5.2 (30) | NA | NA | NA | NA | NA |
| Nayak 2006^29^ | NA | NA | NA | NA | NA | NA |
| Nowak 2009^30^ | 40.3±9.9（35） | 38.9±12.1（35） | NA | NA | 42.2±11.3（41） | 39.6±11.0 (70) |
| Sen 2015^31^ | 53.82±11.17（60） | 56.30±10.84（60） | NA | NA | 55.70±9.82（60） | 55.06±11.03（120） |
| Tomic 2013^32^ | NA | 66.31±8.31（65） | 68.47±7.11（19） | 66.52±7.98（23） | 67.40±4.87（42） | 66.31±8.31（65） |
| Tsunoda 2005^33^ | 61.0±6.9（74） | 63.7±7.8（44） | NA | NA | 65.0±9.8（54） | 62.0±7.3（118） |
| Wang 2010^13^ | 56.6±6.6（45） | 56.2±6.6（49） | 61.1±5.2（46） | 66.2±6.9（41） | 63.5±6.5（87） | 56.4±7.1（94） |
| Yang 2014^34^ | 56.3±8.8（41） | 24.1±3.6（30） | 56.2±7.8（72） | 52.9±10.0 (20) | 55.5±8.6（92） | 56.1±9.2（71） |
| Zorena 2007^35^ | 14±2（41） | 14±3（90） | 15±2（21） | NA | 15±2（21） | 14±2.7（131） |
| Zorena2007^36^ | 15.5±2.2 (35) | 13.08±3.6（70） | NA | NA | 15.72±3.54 (17) | 13.89±3.39（105） |

（23）^#^ 23= number of participants, 44.3±11.6= mean ± SD, SD=Standard Deviation, NA=not available, DM= Diabetes mellitus, NPDR=Non proliferative diabetic retinopathy, PDR= proliferative diabetic retinopathy, Blum 2012^14^ 14= reference number, case= patients with DR, control= diabetic patients without retinopathy and /or matched healthy persons.
