# Supplementary material for: Relationship between C-Reactive Protein Level and Diabetic Retinopathy: A Systematic Review and Meta-Analysis
Source: PLoS One. 2015 Dec 4;10(12):e0144406. doi: 10.1371/journal.pone.0144406 (PMC4670229; doi:10.1371/journal.pone.0144406)
Supplement: S3 Table — (DOCX) [file pone.0144406.s005.docx]

**Sex in all the studies**

| Sex (male : female) Sex (male : female) | | | | | | |
| --- | --- | --- | --- | --- | --- | --- |
| Study | **Healthy** | **DM** | **NPDR** | **PDR** | **Case** | **Control** |
| Blum 2012^14^ | 14:9 (23) | 12:13（25） | 19:6（25） | 13:10（23） | 32:16（48） | 26:24（48） |
| Budak 2013^10^ | 10:14（24） | 14:15（29） | NA | 11:14（25） | 11:14（25） | 24:29（53） |
| Cai 2006^22^ | NA | 51:52（103） | 26:33（59） | 16:12（28） | 42:45（87） | 51:52（103） |
| Chen 2010^11^ | 20:20（40） | 22:23（45） | 22:20（42） | 25:21（46） | 47:41（88） | 42:43（85） |
| Du 2014^16^ | NA | 18:12（30） | 14:9（23） | 9:7（16） | 23:16（39） | 18:12（30） |
| Gho2014^15^ | NA | 32:60 (92) | NA | NA | 40:48 (88) | 32:60 (92) |
| Huang 2006^23^ | 88:116 (204) | NA | NA | NA | NA | NA |
| Jia 2009^12^ | 38:34（72） | NA | NA | NA | NA | NA |
| Kang 2005^24^ | NA | NA | NA | NA | NA | NA |
| Kulkarni 2013^25^ | NA | NA | NA | NA | NA | NA |
| Mastej 2008^26^ | 11:9（20） | 12:10（22） | NA | NA | 19:11（30） | 23:19（42） |
| Mysliwiec 2008^27^ | NA | NA | NA | NA | NA | NA |
| Mysliwska 2012^28^ | 14:16（30） | NA | 11:13（24） | NA | 11:13（24） | NA |
| Nayak 2006^29^ | NA | NA | NA | NA | NA | NA |
| Nowak 2009^30^ | 15:20（35） | 13:22（35） | NA | NA | 17:24（41） | 28:42（70） |
| Sen 2015^31^ | NA | NA | NA | NA | NA | NA |
| Tomic 2013^32^ | NA | 42:23（65） | 8:11（19） | 17:6（23） | 25:27（42） | 42:23（65） |
| Tsunoda 2005^33^ | 38:36（74） | NA | NA | NA | NA | NA |
| Wang 2010^13^ | NA | NA | NA | NA | NA | NA |
| Yang 2014^34^ | 23:18（41） | 17:13（30） | 42:30 (72) | 7:13 (20) | 49:43（92） | 40:31（71） |
| Zorena 2007^35^ | NA | NA | NA | NA | NA | NA |
| Zorena2007^36^ | 17:18（35） | NA | NA | NA | NA | NA |

（23）^#^ 23= number of participants, NA = not available, DM= Diabetes mellitus, NPDR= Non proliferative diabetic retinopathy, PDR= proliferative diabetic retinopathy, Blum 2012^14^ 14= reference number, case= patients with DR, control= diabetic patients without retinopathy and /or matched healthy persons.
