# Supplementary material for: Relationship between C-Reactive Protein Level and Diabetic Retinopathy: A Systematic Review and Meta-Analysis
Source: PLoS One. 2015 Dec 4;10(12):e0144406. doi: 10.1371/journal.pone.0144406 (PMC4670229; doi:10.1371/journal.pone.0144406)
Supplement: S4 Table — (DOCX) [file pone.0144406.s006.docx]

**BMI in all the studies**

| BMI BMI | | | | | | |
| --- | --- | --- | --- | --- | --- | --- |
| Study | **Healthy** | **DM** | **NPDR** | **PDR** | **Case** | **Control** |
| Blum 2012^14^ | 25±4 (23)^#^ | 30±6（25） | 29±4（25） | 30±5（23） | 29.5±4.5（48） | 27.6±5.7（48） |
| Budak 2013^10^ | 26±8（24） | 28±5（29） | NA | 27±6（25） | 27±6（25） | 27±6.5（53） |
| Cai 2006^22^ | NA | 25.10±3.47（103） | 24.60±3.76（59） | 24.45±3.63（28） | 24.55±3.70（87） | 25.10±3.47（103） |
| Chen 2010^11^ | NA | NA | NA | NA | NA | NA |
| Du 2014^16^ | NA | 22.92±1.82（30） | 24.08±2.15（23） | 23.41±2.37（16） | 23.81±2.24（39） | 22.92±1.82（30） |
| Gho2014^15^ | NA | NA | NA | NA | NA | NA |
| Huang 2006^23^ | NA | NA | NA | NA | NA | NA |
| Jia 2009^12^ | NA | NA | NA | NA | NA | NA |
| Kang 2005^24^ | NA | NA | NA | NA | NA | NA |
| Kulkarni 2013^25^ | NA | NA | NA | NA | NA | NA |
| Mastej 2008^26^ | 25.55±1.70（20） | 31.59±5.73（22） | NA | NA | 31.15±3.41（30） | 28.71±5.24（42） |
| Mysliwiec 2008^27^ | 18.34±2.6（85） | 19.75±2.50（163） | NA | NA | 20.52±3.40（39） | 19.27±2.6（248） |
| Mysliwska 2012^28^ | NA | NA | NA | NA | NA | NA |
| Nayak 2006^29^ | 27.5±7.34（44） | 29.1±9.5（44） | NA | NA | 28.7±4.2（30） | 28.3±8.5（88） |
| Nowak 2009^30^ | 22.8±3.2（35） | 22.8±3.2（35） | NA | NA | 20.9±2.1（41） | 22.8±3.2（70） |
| Sen 2015^31^ | 21.3±2.92（60） | 20.33±2.97（60） | NA | NA | 21.00±2.31（60） | 20.8±2.97（120） |
| Tomic 2013^32^ | NA | 30.77±6.06（65） | 30.91±5.28（19） | 30.12±5.33（23） | 30.48±5.26（42） | 30.77±6.06（65） |
| Tsunoda 2005^33^ | 23.0±2.90（74） | 23.2±3.3（44） | NA | NA | 23.6±2.7（54） | 23.1±3.0（118） |
| Wang 2010^13^ | NA | NA | NA | NA | NA | NA |
| Yang 2014^34^ | 23.6±4.1（41） | 24.1±3.6（30） | 23.0±0.5（72） | 23.9±0（20） | 23.2±3.7（92） | 23.8±3.9（71） |
| Zorena 2007^35^ | NA | NA | NA | NA | NA | NA |
| Zorena2007^36^ | NA | NA | NA | NA | NA | NA |

（23）^#^ 23= number of participants, 25± 4= mean ± SD, SD=Standard Deviation, NA = not available, BMI= body mass index, DM=Diabetes mellitus, NPDR= Non proliferative diabetic retinopathy, PDR= proliferative diabetic retinopathy, Blum 2012^14^ 14= reference number, case= patients with DR, control= diabetic patients without retinopathy and /or matched healthy persons
